# Gaming experience affects the interpretation of ambiguous words

**DOI:** 10.1371/journal.pone.0243512

**Published:** 2020-12-28

**Authors:** Rachel B. Eligio, Michael P. Kaschak

**Affiliations:** Department of Psychology, Florida State University, Tallahassee, Florida, United States of America; University of Milan, ITALY

## Abstract

Rodd et al. (2016) report that recreational rowers’ acquisition of sport-related terminology affected their interpretation of words that have both rowing-related and non-rowing-related meanings (e.g., *crab*). The extent to which the rowing- and non-rowing-related meanings were accessible to the participants depended on experiential factors, such as how long the participant had been a rower, and how long it had been since they last rowed. We present two experiments that attempt to replicate these findings with another group of hobbyists, namely video game players. Experiment 1 examined the differences in word meaning choice between gamers and non-gamers. Participation in video-gaming lead to participants generating more gaming-related word meanings in a word association task. Experiment 2 further examined the effects of video gaming experience on the lexical representation of gaming-related words. Participants who had spent more years as gamers were more likely to produce gaming-related word meanings in a word association task. The effect of time spent gaming was no longer significant when we took into account whether the participant engaged with video-game related media (such as YouTube channels or gaming-related message boards). This finding helps us to refine our understanding of the results reported by Rodd et al. (2016), suggesting that it may not be the time spent in an activity that affects the interpretation of ambiguous words, but rather the specific exposure to activity-related vocabulary.

## Introduction

When confronted with an ambiguous word, such as *hand*, comprehenders can access a number of possible meanings. *Hand* can refer to a body part, a set of cards dealt to a poker player, or the transfer of an object from one person to another using the hand, among other possible interpretations. One of these interpretations is the *dominant* interpretation (e.g., hand = body part), whereas the other interpretations are considered to be *subordinate* (e.g., hand = a set of cards). The dominance of an interpretation reflects the frequency of that interpretation in language use, and such interpretations are accessed more quickly during language processing (e.g., [1–3]). Subordinate interpretations occur with lower frequency, and are accessed more slowly (e.g., [1, 2]).

Dominant interpretations of ambiguous words are accessed more readily than subordinate interpretations, though a range of factors affect this general pattern. The speed with which dominant and subordinate meanings are accessed can be affected by learning, prior knowledge, and experience [4–14]. With respect to learning and experience, Hulme et al. [9] showed that adults are able to acquire new meanings for previously non-ambiguous words. Participants were exposed to these novel meanings within a story narrative, and their accuracy in recalling the novel meanings increased with the number of times they encountered the meaning in the story narrative. Furthermore, Coane and Balota [5] found that new word associations resulted in priming comparable to that seen for semantic primes in a lexical decision task. That is, the priming associated with semantically related prime-target pairs such as *library-book* was similar to the priming observed for newly learned pairs with political or pop-culture relevance, such as *face-book*. Finally, it has been shown that the comprehender’s knowledge on a certain topic can affect sense-selection when processing ambiguous words. Wiley et al. [14] provide one demonstration of this effect in a study comparing performance in participants with high and low levels of baseball knowledge. Participants with high baseball knowledge had more difficulty processing ambiguous words when the baseball-related sense of that word was the subordinate sense (i.e., the subordinate meaning competed more strongly with the dominant sense of the word when it was baseball-related).

Over the past decade, Rodd and colleagues (e.g., [4, 8, 13, 15]) have reported a series of experiments showing that both recent and long-term experience can affect the interpretation of ambiguous words. In many of these experiments, participants are given a word, and asked to indicate the first idea that comes to mind when they think about the word (a word association task). As would be expected, participants generally produce a response related to the dominant sense of these words. Rodd and colleagues (e.g. [15]) have demonstrated that a small number of exposures to the subordinate sense of the words is sufficient to raise the odds of producing a response related to the subordinate meaning of a particular word. The priming effects have been shown in the lab [4, 8, 13, 15] and in naturalistic settings [13], have been shown to persist across presentation modalities (e.g., visual or auditory presentation of the words) [8], and are unaffected by changes in speaker identity [13, 15].

Of particular interest for the work reported here was a set of experiments in which Rodd et al. [13] demonstrated that both long- and short-term experience with a specific domain can affect the interpretation of ambiguous words (Experiments 3 and 4 in Rodd et al.’s paper). Rodd et al. [13] examined the role of recent and long-term rowing experience on participants’ interpretation of rowing-related terms (e.g., *crab- malfunction in which the oar is stuck*). When participants were asked to provide associates to ambiguous rowing-related terms, their amount of long-term rowing experience (as measured by a questionnaire about their rowing experience) and recent exposure to the sport (as measured by questions about the number of times the participant had been rowing within the last days, weeks, and month) affected the odds of the participants producing a rowing-related response. Compared to non-rowers, the rowers provided more rowing-related responses to the words. As well, individuals who had rowed the same day as they completed the study gave more rowing meanings than those who had not rowed that day. Finally, individuals with more long-term rowing experience were more likely to provide rowing-related responses.

The results of Rodd et al.’s [13] Experiments 3 and 4 are consistent with the larger body of literature suggesting that experience and expertise can affect the way that word meanings are accessed (e.g., [14, 15]). The experiments also provide two important extensions on this literature. First, Rodd et al.’s [13] work examines the way that different levels of experience–both long- and short-term experience–affect the selection of word meanings. Second, the experiments demonstrate that experiential effects on word processing can be observed outside of the lab. By looking at the interplay between multiple layers of experience in a somewhat naturalistic context, Rodd et al.’s [13] work represents a useful step toward understanding how different sorts of experience might affect language users’ representations of word meaning over time.

On a theoretical level, Rodd et al.’s [13] results point to the conclusion that lexical representations are not fixed. The representations appear to be constantly evolving over time, such that a range of experiential and contextual factors can affect the interpretation that is given to a word at any given time (see [16], for a discussion of the flexible generation of word interpretations, and [11] for a theoretical proposal). The idea that lexical representations are flexible fits within the broader literature on language processing, where there is strong evidence for the malleability of both perceptual (e.g., [17]) and syntactic (e.g., [18, 19]) representations. That is, it seems that language processing in general is characterized by the flexible application of knowledge and contextual constraints to the comprehension task at hand.

Rodd et al.’s [13] Experiments 3 and 4 make an important contribution to the literature on word processing, addressing questions about the short- and long-term temporal aspects of changes in a person’s lexical representations. As these experiments are somewhat unique in the literature (to our knowledge, there are no other published studies that address the malleability of lexical representations on these time scales, and with this sort of methodology), we thought it would be worthwhile to assess the generality of Rodd et al.’s [13] results by conducting a similar study using a different group of hobbyists. Whereas Rodd et al. [13] examined whether sport-related experience affected rowers’ representations of rowing-related terms such as *crab*, we examined whether similar effects would be observed with dedicated video game players. We conducted two experiments using the same online word-association methodology as Rodd et al. [13], and examined the degree to which video game playing experience and participation in video game related social media (e.g., online chats, message boards, live streams of game playing) affects the lexical representations of words such as *camp* (in gaming communities, *camping* refers to sitting and waiting in one position for an opponent or enemy), which have specific interpretations in the context of video game play that are distinct from their more traditional interpretations in language use.

Although rowing and video game play are somewhat different as activities (e.g., video game play is readily accessible and can be done at any time of day, can vary in vocabulary use based on the genre or type of game, and is usually done without the supervision of a coach who may instruct with a universal vocabulary, all of which is different than rowing), we expected that our experiments would produce results similar to those of Rodd et al. [13]. Specifically, we expected to observe that video game players were more likely than non-gamers to provide gaming-related responses to the ambiguous words such as *camp*. We further expected that this tendency would be affected by both short-term (how recently the participant engaged with video games) and long-term (how long, and how often, the participant has played video games) experience. Finally, we expected that the tendency to produce gaming-related responses to words such as *camp* would be related to specific elements of gaming-related experience, rather than video game play *per se*. Elements of game play that might encourage the acquisition of gaming-related vocabulary include engaging in online game play while communicating with one’s partners, and consuming gaming-related media such as gaming live streams. Whereas rowers might acquire rowing-related vocabulary more or less directly from a coach or their peers (e.g., an examination of rowing-related periodicals and internet message boards suggests that terms such as *catch* and *drive* are universal labels for parts of the rowing stroke, and would be used as such by coaches), gamers are unlikely to have such direct instruction in activity-related terminology. Gamers are more likely to pick up the terminology implicitly or incidentally while engaging in game play (or consuming gaming-related media).

## Experiment 1

Experiment 1 was designed to examine whether video gaming experience changed the lexical representations of words such as *camp*, such that the (subordinate) gaming-related interpretation would be selected rather than the more traditional, dominant sense of the word. Gamers and non-gamers performed the same word-association task as used by Rodd et al. [13], and we observed whether gamers produced more gaming-related responses to the gaming-related words than non-gamers. We also administered questionnaires to gather information about the participants’ gaming experiences (if any) and assessed their knowledge of the gaming-related meanings of the words used in the experiment.

## Materials and methods

### Participants

We collected data from 146 participants from Florida State University's psychology research pool. The participants were native speakers of English. Participants received course credit or extra credit in exchange for their participation. Data for this experiment was collected in the summer of 2019. Of the 146 participants, there were 58 gamers (21 female, 37 male; aged 18–25; mean age = 18.6 years) and 88 non-gamers (84 females, 4 males; aged 18–22; mean age = 18.5 years).

### Ethics approval

The research protocol used in these experiments was approved by the Florida State University IRB. Consent was not obtained since the data was analyzed anonymously. Written consent was not required by the ethics committee and participants saw an information sheet before the study.

### Materials

Twenty target gaming words (*boss*, *camp*, *cereal*, *cheese*, *farm*, *feed*, *health*, *instance*, *mule*, *patch*, *platform*, *sandbox*, *sheep*, *spawn*, *stream*, *tank*, *troll*, *twitch*, *wave*, *wipe*) were selected from online searches on gaming terminology [20–23] and by consulting with gamers known to the investigators that had 15 years of playing experience (*see* S1 Appendix). Words were selected such that the gaming-related interpretation was distinct from the non-gaming interpretations of the word. In addition to the gaming terms, 78 filler unambiguous words were included. These were taken from the materials of a previous study [24].

### Procedure

The experiment was conducted online, using the Qualtrics survey platform. The experiment consisted of 3 parts: 1) word association task, 2) questionnaire on gaming activity participation, and 3) a gaming word knowledge test. Participants were informed that the purpose of the study was to examine word association and memory. The word association task consisted of 98 words, 78 filler and 20 target gaming terms. Participants saw the words one at a time in a random order and were instructed to provide the first word that comes to mind that is related in meaning to the word presented. Following the word association task, participants were given a questionnaire that asked for demographic information (gender, age, and whether they were a native English speaker) and asked the participants about their participation in different activities. They were presented with 3 groups of activities: group A (writing, painting/drawing, reading, photography, pottery/sculpting), group B (social media, watching movies, watching tv, gaming, listening to music), and group C (baseball/softball, football, soccer, tennis, and disc golf). Within each category, they were asked questions on participation (yes/no), length of participation (years, days since last participated, average times in a month), type of participation (alone or in a group), and if they watch media related content (tv, online) on the activity. Following the activity questions, participants were asked if they knew the aim of the study. For the final phase of the experiment, participants were given a word knowledge task on the 20 gaming terms that were presented in random order. They were asked to indicate if they were familiar with the gaming-related sense of each word (yes/no). If they indicated that they knew the meaning of the word, they were asked to provide the definition of the word. Finally, participants were presented with gaming questions that were intended to collect additional information on gaming exposure. These questions included how they came to learn the known gaming definitions (online gaming with others, in person gaming with others, watching media on gaming, online chat boards, and so on), whether or not they use gaming terms, where they have used gaming terms (online gaming with others, in person gaming with others, online chat boards, streaming online game play), what type of gamer that they consider themselves (non-gamer, novice, casual, hardcore, or professional), if their game playing has been monetized, and the type of games that they prefer (action, role-playing, sports, strategy, puzzle video games, no preference).

### Coding

Responses to the word association task and the word knowledge task were coded by RE. The coder was blind to identity of the participants and whether they identified as a gamer or not when performing the coding. For each word, the investigators generated a general gaming-related definition that captured the gist of how the word was used within the gaming community. Participant responses were coded as being gaming-related if they captured the core of the experimenter-generated definition.

Participants were coded as gamers based on their responses to the gaming activity questionnaire. Participants who indicated that they participated in gaming of any type were coded as gaming. Otherwise, they were coded as non-gamers.

### Data analysis

We analyzed the data with a series of mixed models logistic regressions aimed at predicting the log odds of producing a gaming-related meaning for the 20 critical items used in the word association task. The dependent measure was a binary coding of the participant responses as a gaming-related response (coded as 1) or a non-gaming-related response (coded as 0). Following Rodd et al. [13], we examined each participants’ performance on the test of gaming word knowledge, and only included items for which the participant indicated that they knew the meaning of the gaming word and provided a correct definition. In this experiment, participants coded as gamers knew an average of 7.84 of the gaming words included on our list, and non-games knew an average of 3.08 of the gaming words on our list. Thus, the dependent measure could address the question of whether the participant produced the gaming-related meaning of the word in the word association task, given that they knew the meaning of the gaming word.

The first mixed models logistic regression assessed whether gamers were more likely to produce gaming-related responses than non-gamers. Our binary coding of the participant responses was the dependent measure, and the predictor was Gamer (non-gamers coded as 0, and gamers coded as 1). Participants and items were included as crossed random factors. Due to convergence issues, random by-item slopes were not included in the model. Note that our elimination of trials on which participants did not demonstrate knowledge of the gaming-related word on the post-test meant the elimination of much of the trials from some of the participants (particularly the non-gamers), and eliminated all of the data for some participants. With the elimination, 2,462 trials and 56 people were removed (the participants were removed because all of their trials were eliminated on account of their word-knowledge test performance). Although we report the results of this analysis below, we also conducted a parallel analysis that included all data from the word association task (i.e., we did not exclude trials for which participants did not know the word on the word knowledge test). This analysis produced results that were essentially the same as for the primary analysis (*see* Table 1b in S2 Appendix).

Our second mixed models logistic regression analysis explored the effects of gaming experience on the odds of producing a gaming-related response on the word association test. Because our interest is in looking at variation in gaming experience, we limited this analysis to the participants coded as gamers. Our binary coding of the word association responses was the dependent measure, and the predictors were age and number of years of playing video games (Years of Gaming; measuring long-term gaming experience), average number of times playing video games per month (Gaming per Month; measuring medium-term experience), and number of days since last playing video games (Days since Last Played; measuring short-term experience). Participants and items were included as crossed random factors. Due to issues with model convergence, random by-item slopes were not included in the model. Similar to Rodd et al.’s [13] initial study, all temporal variables were all log transformed before being entered into the model. One participant indicated having played video games from birth (i.e., the age listed was the same as the number of years playing video games). This struck us as implausible, so we capped the number of years playing video games at 15 years (which, given the age of our participants, would mean they started playing video games around preschool or kindergarten age). In addition, one participant indicated having played video games 999 times per month. This similarly struck us as implausible, so we capped the number of times playing video games per month at 100. We ran another analysis without these caps in place, and they produced essentially the same results. Similar to the first analysis, we conducted a parallel analysis that included all the responses from the word association task. The results were similar aside from the predictor Days Since Last Played (*see* Table 2b in S2 Appendix)

Our final mixed models logistic regression assessed whether gamers were more likely to produce gaming-related meanings based on aspects of their gaming activity. The binary coding of word association responses was again the dependent measure, and the predictors were Type of Participation (solo game play coded as -1, playing in groups coded as 1) and Use of Gaming Terms (no use of gaming terms coded as -1, use of gaming terms coded as 1). Use of Media (using gaming-related media coded as 1, no use of media coded as -1) was not analyzed due to a lack of variability (essentially all gamers reported using gaming-related media). Participants and items were included as crossed random factors. Due to issues with model convergence, random by-item slopes were not included in the model. Finally, a parallel analysis of all responses from the word association task was conducted and produced similar results to the primary analysis (*see* Table 4b in S2 Appendix).

All analyses were run with the glmertree library [25] in R Studio [26]. The data and code for these analyses, as well as all materials associated with the experiment, are available on the Open Science Framework (https://osf.io/ua238/).

## Results

The first question addressed in our analyses was whether gamers would produce more gaming-related responses to the word association task than non-gamers. The results of a mixed models logistic regression predicting the log odds of producing a gaming-related response is shown in Table 1. There was a significant difference (*p* = .023) in the proportion of gaming-related responses produced by gamers (M = 0.280, SD = 0.338) and the proportion of such responses produced by non-gamers (M = 0.146, SD = 0.301). As expected, gamers produced more gaming-related responses than non-gamers.

**Table 1:** Experiment 1: Mixed logit analysis of participant gaming responses.

Predictor	Coefficient	SE	*z*-value	*p*-value
Intercept	-3.471	0.612	-5.669	< .001
Gamer	0.897	0.395	2.268	0.023

N = 90, 458 total observations.

The results of our second analysis, examining whether the odds of producing a gaming-related response in the word association task was predicted by Age, and by long- (Years of Gaming), medium- (Gaming per Month), and short-term (Days since Last Played) gaming experience, are presented in Table 2. This analysis included data from the gamers, and excluded non-gamer data. None of the temporal variables included in the model were significant predictors of the participants’ likelihood of producing a gaming-related response. In addition, descriptive statistics for each amount of experience predictor variable were examined and presented in Table 3.

**Table 2:** Experiment 1: Mixed logit analysis of participant gaming responses predicted by gaming experience.

Predictor	Coefficient	SE	*z*-value	*p*-value
Intercept	1.591	17.355	0.092	0.927
Years of Gaming	2.009	1.414	1.421	0.155
Age	-5.451	13.574	-0.402	0.688
Gaming per Month	0.475	0.633	0.751	0.453
Days since Last Played	0.663	0.389	1.704	0.088

N = 38, 298 total observations.

**Table 3:** Descriptive statistics for predictor variables in mixed logit analyses for gamers in experiment 1 and 2.

	Age(years)	Years of Gaming	Gaming per Month(times)	Days Since Last Played
Experiment 1				
Min	18	1	0	0
Max	25	15	100	100
Mean	18.55	10.64	19.03	8.43
Experiment 2				
Min	18	1	0	0
Max	28	15	100	7
Mean	19.62	8.01	10.81	1.74

A final set of analyses assessed whether the gamer’s Use of Gaming Terms, and whether or not their Type of Participation, predicted the odds of producing a gaming-related response on the word association task. The results of this analysis are presented in Table 4. Neither variable was a significant predictor of the production of gaming-related responses in the word association task.

**Table 4:** Experiment 1: Mixed logit analysis of participant gaming responses predicted by use of gaming terms and type of participation.

Predictor	Coefficient	SE	*z*-value	*p*-value
Intercept	-2.624	0.59	-4.45	< .001
Use of Gaming Terms	0.127	0.311	0.409	0.682
Type of Participation	-0.0196	0.265	-0.074	0.941

N = 37, 296 total observations.

We asked participants whether they were aware of the purpose of this study. Only one participant correctly guessed the purpose of the study. We therefore re-ran the analyses excluding this participant. The pattern of significant and non-significant results was identical to what was in the analyses reported above.

## Discussion

Our results partially replicate the results of Rodd et al.’s [13] study. Participation in a recreational activity with a specialized use of particular words (video gaming) had a reliable effect on the interpretation of ambiguous words. In contrast to Rodd et al.’s [13] results, the effects of recent, medium, and long-term experience did not significantly affect the odds of producing a gaming-related response to the word association task among those participants identified as gamers. A possible explanation for this null result is that our definition of gamers was somewhat broad, including individuals who engaged in a wide range of game activities. Playing some types of games (e.g., multi-player combat games) would make it more likely that the participant would have been exposed to the gaming words used in our study, whereas playing other games (e.g., word games) would make it unlikely that the participant would have been exposed to the gaming words. In this case, knowledge of the gaming words would depend more on the games that the participant played than on the amount of time they spent playing games. A second issue is that our time-based measures (particularly the measures of short-term experience) were somewhat broad. A third issue is that our exclusion of word association trials based on the results of our participants’ word knowledge test left a low number of items (~ 5 items) per participant, reducing the precision and power of the study. Related to power, a final issue is that due to our sampling plan (sampling participants in general, then separating them out into gamers and non-gamers), our final sample size for the analyses involving only gamers was somewhat small (*n*’s < 50) and likely under-powered. These potential issues were rectified in Experiment 2.

## Experiment 2

Experiment 2 was designed to replicate and extend Experiment 1. The experiment used the same structure as Experiment 1 (word association task, followed by a questionnaire, followed by the word knowledge test and a few additional questions). To increase the odds that our participants would have at least encountered the gaming words used in the word association task, we recruited participants from our local participant pool specifying that we were interested in recruiting individuals who specifically engaged in video game activities. In addition, we adapted our questions about the frequency and duration of video game playing to get finer-grained information about recent gaming experience. Finally, we increased our sample size and restricted our sample to gamers only to improve our ability to detect the effects of our predictors on the production of gaming-related word meanings. These changes to the design were aimed at providing a better opportunity to observe the type of time-based effects reported by Rodd et al. [13].

## Materials and methods

### Participants

The participants were 197 undergraduate students from the psychology research pool at Florida State University. The recruitment materials suggested that this was an experiment about word associations and memory. It was further specified that participants should be video gamers, though we did not provide a definition of video gamers. None of these participants had taken part in the Experiment 1. All participants were native English speakers. They received course credit or extra credit in exchange for their participation. We excluded 4 participants for not finishing the survey and an additional 2 participants were excluded because they listed zero years of gaming experience. The final sample was 191 participants (114 females; 77 males; aged 18–28 years; mean age = 19.6 years).

These data were collected between November 2019 and December 2019. [Note: We also planned to recruit participants from gaming-related message boards on Reddit. Because of a low overall response to our recruiting (n < 25), and the failure of most participants to complete the entire study, we decided to exclude this sample from the analyses reported here].

### Materials

The stimuli were the same as in Experiment 1 (*see* S1 Appendix).

### Procedure

The procedure was largely the same as in Experiment 1. Participants completed the word association task, followed by a questionnaire asking for demographic information. For this experiment, the questionnaire included questions about age, gender, and whether they were a native speaker of English. At the end of the questionnaire, we asked participants whether they knew what the goal of the study was. Following the aim question, the test of gaming word knowledge and a questionnaire about gaming experience were presented. The gaming questionnaire contained the same questions as Experiment 1, an additional question on which video game that they play the most, and the detailed experience questions. As in the last experiment, participants were asked how many years they had participated in video-gaming. Following Rodd et al.’s [13] Experiment 4, participants were asked to indicate which day they were completing the survey. From their response, they received one of 7 possible variations. For example, if they checked Monday, they would see a question indicating for them to check all the times in 2 hour increments that they had participated in video-gaming over the past week from Monday back to last Sunday. Following this question, participants were asked to indicate the number of average hours that they engaged in video gaming per week for the last 10 months. The last 10 months ranged from October back to January.

### Coding

The responses to the word association task and the gaming word knowledge task were coded as in Experiment 1.

### Data analysis

The data were analyzed with a series of mixed models logistic regressions. The dependent measure in each of these analyses was the binary coding of the participants’ responses to the word association task (non-gaming-related responses coded as 0, gaming-related responses coded as 1). As in Experiment 1, we excluded word association trials for any words that individual participants did not define correctly in the word knowledge test. Participants defined an average of 8.19 words correctly on this test. There were 2,723 trials excluded, and the data from 57 participants excluded (the participants were removed because all of their trials were eliminated on account of their word-knowledge test performance).

The first analysis used our time-based measures to predict the likelihood of producing a gaming-related response in the word association task. The dependent measure was the binary coding of the participants’ word association responses. Our original plan was to use the following predictors: age, number of years of playing video games (Years of Gaming; long-term measure of gaming experience, again capped at 15 years), number of times playing video games over the past month (Gaming per Month), average number of times playing video games per month over the last 9 months, number of days since last playing video games (Days since Last Played; as in Rodd et al., 2016 [18], this was capped at 7), and the number of times playing video games in the last week. The number of times playing video games over the past month, and the average number of times playing video games per month over the last 9 months were considered medium-term measures of gaming experience. The number of times playing video games in the last week, and the number of days since last playing video games were considered short-term measures of gaming experience. The predictors were log transformed prior to the analysis. A model containing all of these variables would not converge, so we ran a simpler model with the following predictors: age, number of years since playing video games (Years of Gaming), average number of times playing video games per month over the last nine months (Gaming per Month), and number of days since last playing video games (Days since Last Played). That is, we eliminated one medium-term and one short-term measure of gaming experience from the model. We elected to eliminate these variables because they were significantly correlated with the other medium-term and short-term variables, and we kept the variables that had higher raw correlations with the dependent variable. Participants and Items were included as crossed random factors in the model. Due to convergence issues, random by-item slopes were not included in the model. In addition, a parallel analysis was conducted for all words in the word association task and the results were similar to the primary analysis except for predictor Age (*see* Table 5b in S2 Appendix).

We conducted a further set of mixed models regression analyses aimed at exploring the influence of a broader set of variables on participants’ likelihood of producing a gaming-related response in the word association task. The variables Use of Media (no use of media coded as -1, use of media coded as 1), Type of Participation (solo game play coded as -1, playing in groups coded as 1), and Use of Gaming Terms (no use of gaming terms coded as -1, use of gaming terms coded as 1). We initially planned to include all three variables in a regression model along with the experiential variables included in the mixed models regression analysis described above. This model would not converge, so we ran three mixed models regressions that had the experiential variables described above, plus one of the three additional variables (Use of Media, Type of Participation, and Use of Gaming Terms) of interest. Participants and Items were crossed random factors in each of the analyses. Due to convergence issues, random by-item slopes were not included in these models. Additional analyses were also conducted on all words in the word association task and revealed some discrepant results for each of the three mixed models (*see* Table 6b in S2 Appendix).

The data and code for the analysis of Experiment 2, as well as the materials associated with this experiment, are available on OSF (https://osf.io/ua238/).

## Results

Our primary analysis addressed the question of whether Age, long-term (Years of Gaming), medium-term (Gaming per Month), and short-term experience (Days since Last Played) with video gaming predicted the log odds of producing a gaming-related response in the word association task. This model is presented in Table 5. The only significant predictor was the Years of Gaming (*p* = .007). Participants with more years of gaming experience were more likely to produce a gaming-related meaning on the word association task.

**Table 5:** Experiment 2: Mixed logit analysis of participant gaming responses predicted by gaming experience.

Predictor	Coefficient	SE	*z*-value	*p*-value
Intercept	3.232	4.566	0.708	0.479
Years of Gaming	1.35	0.5	2.7	0.007
Age	-5.425	3.545	-1.531	0.126
Gaming per Month	0.14	0.207	0.677	0.499
Days since Last Played	-0.086	0.058	-1.486	0.137

N = 134, 1097 total observations.

We next assessed a series of models where we added individual predictors (Use of Media, Type of Participation, and Use of Gaming Terms) to the model presented in Table 4. These models are presented in Table 6. In the first model, Use of Media was a significant predictor of producing a gaming-related response in the word association task (*p* < .001). Participants who used gaming-related media were more likely to produce a gaming-related meaning in the word association task than those who did not use such media. None of the experiential variables were significant predictors in this model. In the second model, Type of Participation was not a significant predictor of producing a gaming-related response. However, the Years of Gaming was a significant predictor (*p* = .007). In the third analysis, the Use of Gaming Terms and the Years of Gaming approached significance. None of the other experiential variables were significant predictors in this model.

**Table 6:** Experiment 2: Mixed logit analyses of participant response predicted by gaming experience, use of media, type of participation, and use of gaming terms.

Predictor	Coefficient	SE	z-value	p-value
Intercept	3.302	4.432	0.745	0.456
Years of Gaming	0.745	0.507	1.47	0.141
Age	-5.257	3.439	-1.529	0.126
Gaming per Month	0.099	0.194	0.512	0.608
Days since Last Played	-0.065	0.056	-1.159	0.246
Use of Media	0.579	0.158	3.675	< .001
Intercept	3.248	4.566	0.711	0.477
Years of Gaming	1.353	0.501	2.7	0.007
Age	-5.437	3.545	-1.534	0.125
Gaming per Month	0.139	0.207	0.671	0.503
Days since Last Played	-0.087	0.058	-1.489	0.136
Type of Participation	-0.012	0.123	-0.099	0.921
Intercept	2.19	4.586	0.477	0.633
Years of Gaming	1.004	0.534	1.879	0.06
Age	-4.497	3.566	-1.261	0.207
Gaming per Month	0.145	0.206	0.705	0.481
Days since Last Played	-0.08	0.058	-1.362	0.173
Use of Gaming Terms	0.29	0.162	1.797	0.072

N = 134, 1097 total observations.

Based on the results of the following analyses, we analyzed the correlations between Years of Gaming, Use of Media, and Use of Gaming Terms as presented in Table 7. The predictor Years of Gaming was significantly correlated with Use of Media, *r* = 0.275. In addition, the predictor Use of Gaming Terms was significantly correlated with Years of Gaming, *r* = 0.390 and Use of Media, *r* = 0.286.

**Table 7:** Experiment 2: Correlations between gaming experience, use of media, and use of gaming terms.

Measure	1	2	3
1. Years of Gaming	___		
2. Use of Media	0.275**	___	
3. Use of Gaming Terms	0.390**	0.286**	___

**Correlation is significant at 0.01 level (2-tailed).

Finally, we asked whether participants were aware of the purpose of the experiment. Thirty-one participants correctly guessed the purpose of the experiment. We re-ran the analyses in Tables 5 and 6 excluding these participants, but the models did not converge. As a result, the data was analyzed using multiple regression analyses. The dependent variable was the average number of gaming related responses produced by each participant. Again, the predictor variables were log-transformed. The models are presented in Tables 8 and 9. The results (with regard to significant and non-significant predictors) were similar for the results of the analyses reported in Tables 5 and 6.

**Table 8:** Experiment 2: Multiple regression of participant response predicted by gaming experience.

Predictor	Coeff	SE	*t-*value	*p-*value
Constant	0.776	0.688	1.128	0.262
Years of Gaming	0.166	0.064	2.581	0.011
Age	-0.574	0.531	-1.08	0.283
Gaming per Month	0.015	0.026	0.58	0.563
Days since Last Played	-0.004	0.009	-0.474	0.637

**Table 9:** Experiment 2: Multiple regression of participant response predicted by gaming experiences, use of media, type of participation, and use of gaming terms.

Predictor	Coeff	SE	*t*-value	*p*-value
Constant	0.491	0.67	0.733	0.465
Years of Gaming	0.053	0.071	0.743	0.459
Age	-0.298	0.520	-0.572	0.568
Gaming per Month	0.013	0.025	0.504	0.615
Days since Last Played	-0.002	0.009	-0.195	0.846
Use of Media	0.046	0.022	2.099	0.038
Type of Participation	-0.023	0.019	-1.213	0.228
Use of Gaming Terms	0.045	0.022	2.066	0.041

## Discussion

When examining whether different ranges of experience predict the odds of a participant generating a gaming-related response in our word association task, only long-range experience was a significant predictor of the participant making gaming-related responses. Our subsequent analyses showed that the Use of Media was a strong predictor of producing gaming-related responses in the word association task. This is presumably because the Use of Media would increase the odds of being exposed to particular gaming terms, and the frequency with which the participant encountered the terms. Notably, once Use of Media was included in the regression models, none of the experiential variables were significant predictors of the production of gaming-related responses. The predictor Use of Gaming Terms also approached significance when included in our main analyses, and was significant in the multiple regression. The fact that both Use of Media and Use of Gaming Terms were significant (or marginally significant) predictors in these analyses highlights the fact that it may not be experience (as such) that leads to knowledge of gaming terms, but rather specific kinds of experience that are likely to expose the gamer to the relevant vocabulary. More generally, the fact that both variables are significant when included in the same model suggests that they play different roles in driving the production of gaming-related meanings in the word association task: Use of Media may index the exposure needed for participants to be aware of the gaming-related meanings of the target words, and Use of Gaming Terms may index the increased learning of these meanings that come with using the words yourself.

## General discussion

Rodd et al. [13] used an online survey method to examine the role of experience in shaping the interpretation of ambiguous words. They found that the interpretation of ambiguous rowing-related words such as *crab* was affected both by long-term (how many years had the participant been rowing) and short-term (did the participant row on the day of the experiment?) experience. We conducted two experiments aimed at establishing the generality of these results by using a similar online survey method to study the role of experience in shaping video game players’ interpretation of ambiguous gaming-related words, such as *camp*. In Experiment 1, we found that gamers produced more gaming-related responses than non-gamers. In Experiment 2, and similar to Rodd et al. [13], we found that the number of years that our participants had been playing video games (i.e., long-term experience) was a significant predictor of producing gaming-related responses on the word association task. However, long-term experience did not significantly predict the production of gaming-related terms once we accounted for the participants’ use of gaming-related media. These results suggest that it may not be the amount of experience with video gaming that is driving the production of gaming-related responses, but rather the type of experience the participants have with video games–specifically, experiences such as using gaming-related social media that allow the participant to engage with gaming-related vocabulary. Those participants who engaged with gaming-related social media were more likely to produce gaming-related responses. Our findings put an important qualification on Rodd et al.’s [13] results, and suggest that future investigations of this topic should include both time-based measures of engagement with the target activity and more direct measures of the participants’ exposure to activity-related vocabulary.

Another way in which our results diverge from Rodd et al.’s [13] findings is that we did not find evidence that short-term experience (e.g., whether the participant engaged in the target activity on the day of the study) affects the production of gaming-related terms. We do not have a ready explanation for the lack of a short-term experience effect in our study. One possibility is that gaming terms may be less contextually-bound than rowing terms, and their availability in memory may therefore be less dependent on engaging in the target activity. For example, the rowing-related meanings of *crab*, *catch*, and other such terms may only occur in a rowing context, and therefore participating in rowing activities would boost their availability. However, the gaming-related meaning of terms such as *camp* could be used in a broader context (e.g., just as someone *camps* to await opponents in a video game, a person selling products in a store might *camp* waiting to ambush customers with their product), making it less likely that gaming-related activities would be needed to activate the gaming-related meaning of the word. Thus, a gamer might be more likely than a rower to have activity-related terms creep into their general use of language.

The results of our experiments fit with Rodd et al.’s [13] general conclusion that experience within a particular domain can shape the interpretation of ambiguous words (see [14], for another demonstration of this effect). The cases where our results diverged from theirs are likely due to differences in the nature of our samples, and differences in the nature of the activities that we examined. For example, Rodd et al. [13] report that age is a significant (negative) predictor of producing rowing-related responses in their sample, but we found no effects of age. The age range in our samples was much smaller than that in Rodd et al.’s [13] samples (age range is 18–25 in our samples, and 17–55 in Rodd et al.’s samples), and this restriction of range may have affected our ability to observe an age effect. The range of years engaged in the target activity (gaming or rowing) was also smaller in our sample (1–15 years in our samples, and 0–42 years in Rodd et al.’s samples).

Our finding that use of media, and not experience per se, is the driving force behind participants producing gaming-related responses may result from differences between rowing and video game play as activities. Rowing is an organized sport, and it is likely that all who participate are introduced to the same rowing-related vocabulary (e.g., *feathering* the blade, catching a *crab*). That is, anyone who has spent time learning the sport was likely exposed to the rowing-related terms used by Rodd et al. [13]. In contrast, video gaming is a less-organized activity, and the use of particular gaming-related terms is not as uniform across individuals who engage in this activity. For example, *camping* (staying in the same place and hiding in wait for your enemies) is a term that is relevant to combat-related games such as *Call of Duty*, but not relevant to other types of popular games, such as *Animal Crossing* or sports-related games. In addition, games such as *Call of Duty* can be played in a solo format whereas, games such as *League of Legends* and *World of Warcraft* may be played in a group setting and use different gaming vocabulary. It is therefore likely that gamers need exposure to specific other gamers, or to social media, in order to be exposed to the specific gaming-related terminology that has developed around certain kinds of games. More broadly, the diversity of gaming-related terms across games and gaming sub-cultures may mean that any particular episode of gaming may or may not result in hearing a particular gaming-related term (whereas the standardized vocabulary of rowing makes it more likely that a rower will her specific rowing-related terms while engaging in the activity). This may be another possible explanation for why short-term experience did not affect the production of gaming-related responses in our experiments (see earlier discussion).

An important limitation to our findings is the potential lack of statistical power in both experiments. Experiment 1 likely suffered from both having a small sample of gamers and from having a small number of trials per participant (due to screening items based on the post-test responses). Experiment 2 had a larger sample size, but still suffered from power issues due to the small number of trials per participants (as in Experiment 1). The loss of trials in our experiments is likely driven by the fact that the lexical items related to video games is variable across gaming communities (unlike rowing, which likely has a standard vocabulary that is widespread in the sport). Thus, whereas we believe that our main findings (gamers produce more gaming-related definitions than non-gamers, and gamers’ use of gaming-related terms is driven by their exposure to the terminology through various avenues) are likely to be correct, these conclusions should be verified through additional research that targets specific gaming communities (and their specialized lexicon) more directly.

Our data contribute to the literature showing that different kinds of experience affect the interpretation of ambiguous words (e.g., [11, 13–15]), as well as to the broader literature that highlights the role of experience in language processing (e.g., [17–19, 27]). As Rodd [11] argues, the flexibility with which we interpret words, and the time-course that governs the malleability of lexical representations, places important constraints on our theories of lexical representation and meaning. Although the interplay between the impact of experience over short (e.g., [15]) and longer (e.g., [13]) spans of time is likely to be complicated, the extant literature provides an important start toward untangling these relationships.

## Supporting information

S1 Appendix Gaming-related terms, definitions (D), acceptable associations (AA) and non-acceptable associations (NA). (DOCX)

S2 Appendix Analyses with all words in word association task. (DOCX)
