# Supplementary material for: Gaming experience affects the interpretation of ambiguous words
Source: PLoS One. 2020 Dec 28;15(12):e0243512. doi: 10.1371/journal.pone.0243512 (PMC7769253; doi:10.1371/journal.pone.0243512)
Supplement: S1 Appendix — (DOCX) [file pone.0243512.s001.docx]

**S1. Appendix**

**Gaming-related terms, Definitions (D), Acceptable Associations (AA) and Non-acceptable Associations (NA).**

Boss

D: difficult enemy at the end of a game

AA : strong, difficult, end of game, loot

NA: employee, work, company

Camp

D: to sit and wait, not moving for an opponent

AA: hide, wait, position, not moving

NA: campfire, smores, vacation

Cereal

D: serious

AA: serious

NA: food, breakfast

Cheese

D: unforeseen win with little effort, not intended by the developer of the game

AA: cheap, cheat, little effort

NA: food, mouse

Farm

D: repetitive gameplay to receive more items/experience

AA: camp, repetitive, experience, items

NA: animals, crops,

Feed

D: to die to other players so that they receive experience or gold

AA: die, lose, kill, gold/experience

NA: give food, newsfeed

Health

D: health, damage one can take, hit points

AA: stamina, hearts, HP, hit point, damage available

NA: healthy, medical, body

Instance

D: dungeon or separate area for group/single player

AA: dungeon, separate, group area

NA: occurrence, example

Mule

D: mobile bank character

AA: bank, loot, mobile bank character

NA: animal, donkey

Patch

D: update

AA: update, new, improve

NA: clothing, hole, cloth

Platform

D: console or system that game is played on

AA: console, system, (xbox, wii, playstation, computer)

NA: raised surface to stand on,

Sandbox

D: open world

AA: open, open world, creative

NA: sand, playground

Sheep

D: crowd control, polymorph

AA: CC(crowd-control), polymorph

NA: fluffy, animal, white

Spawn

D: to generate/regenerate or starting point in a game

AA: start, appear, generate, regenerate, starting point, create objects

NA: fish, frog eggs

Stream

D: video/audio game content to internet

AA: twitch, watch, internet, video-game content

NA: water

Tank

D: character that takes high damage

AA: tough, defense, damage, character

NA: war, machine

Troll

D: player that troubles/harasses others in the game

AA: bother, trouble, harass

NA: creature, bridge

Twitch

D: website for streaming gameplay

AA: stream, website, gameplay

NA: nervous, eye

Wave

D: group of enemies

AA: event, level, enemies/minions

NA: hand, water

Wipe

D: to die or be killed in the game

AA: die, killed, party kill

NA: clean
