# Supplementary material for: Gaming experience affects the interpretation of ambiguous words
Source: PLoS One. 2020 Dec 28;15(12):e0243512. doi: 10.1371/journal.pone.0243512 (PMC7769253; doi:10.1371/journal.pone.0243512)
Supplement: S2 Appendix — (DOCX) [file pone.0243512.s002.docx]

**S2. Appendix**

**Analyses with all Words in Word Association Task.**

**Table 1b. Experiment 1: Mixed Logit Analysis of Participant Gaming Responses.**

| Predictor | Coefficient | SE | *z*-value | *p*-value |
| --- | --- | --- | --- | --- |
| Intercept | -5.623 | 0.666 | -8.437 | <.001 |
| Gamer | 1.312 | 0.259 | 5.062 | <.001 |
|  |  |  |  |  |
| N=146, 2920 total observations | | | |  |

**Table 2b. Experiment 1: Mixed Logit Analysis of Participant Gaming Responses Predicted by Gaming Experience.**

| Predictor | Coefficient | SE | *z*-value | *p*-value |
| --- | --- | --- | --- | --- |
| Intercept | 4.097 | 8.330 | 0.492 | 0.623 |
| Years of Gaming | 1.305 | 0.818 | 1.596 | 0.111 |
| Age | -7.610 | 6.622 | -1.149 | 0.251 |
| Gaming per Month | 0.010 | 0.209 | 0.046 | 0.963 |
| Days since Last Played | 0.329 | 0.150 | 2.201 | 0.028 |
|  |  |  |  |  |
| N=58, 1160 total observations | | | |  |

**Table 4b. Experiment 1: Mixed Logit Analysis of Participant Gaming Responses Predicted by Use of Gaming Terms and Type of Participation.**

| Predictor | Coefficient | SE | *z*-value | *p*-value |
| --- | --- | --- | --- | --- |
| Intercept | -4.323 | 0.676 | -6.399 | <.001 |
| Use of Gaming Terms | 0.280 | 0.192 | 1.461 | 0.144 |
| Type of Participation | 0.145 | 0.177 | 0.820 | 0.412 |
|  |  |  |  |  |
| N=56, 1120 total observations | | |  |  |

**Table 5b. Experiment 2: Mixed Logit Analysis of Participant Gaming Responses Predicted by Gaming Experience.**

| Predictor | Coefficient | SE | *z*-value | *p*-value |
| --- | --- | --- | --- | --- |
| Intercept | 7.917 | 4.198 | 1.886 | 0.059 |
| Years of Gaming | 2.401 | 0.402 | 5.967 | <.001 |
| Age | -10.710 | 3.257 | -3.289 | 0.001 |
| Gaming per Month | 0.119 | 0.186 | 0.636 | 0.525 |
| Days since Last Played | -0.022 | 0.051 | -0.438 | 0.661 |
|  |  |  |  |  |
| N=191, 3820 total observations | | | |  |

**Table 6b. Experiment 2: Mixed Logit Analyses of Participant Response Predicted by Gaming Experience, Use of Media, Type of Participation, and Use of Gaming Terms.**

| Predictor | Coefficient | SE | z-value | p-value |
| --- | --- | --- | --- | --- |
| Intercept | -5.774 | 0.581 | -9.935 | <.001 |
| Years of Gaming | 2.032 | 0.408 | 4.983 | <0.001 |
| Gaming per Month | 0.072 | 0.182 | 0.394 | 0.694 |
| Days since Last Played | -0.029 | 0.051 | -0.571 | 0.568 |
| Use of Media | 0.386 | 0.141 | 2.742 | 0.006 |
| Intercept | 7.997 | 4.244 | 1.884 | 0.060 |
| Years of Gaming | 2.414 | 0.403 | 5.987 | <0.001 |
| Age | -10.764 | 3.293 | -3.269 | 0.001 |
| Gaming per Month | 0.114 | 0.187 | 0.610 | 0.542 |
| Days since Last Played | -0.024 | 0.051 | -0.474 | 0.636 |
| Type of Participation | -0.086 | 0.110 | -0.785 | 0.432 |
|  |  |  |  |  |
| Intercept | 6.300 | 4.132 | 1.525 | 0.127 |
| Years of Gaming | 1.913 | 0.412 | 4.637 | <0.001 |
| Age | -9.204 | 3.209 | -2.868 | 0.004 |
| Gaming per Month | 0.071 | 0.181 | 0.392 | 0.695 |
| Days since Last Played | -0.007 | 0.050 | -0.134 | 0.893 |
| Use of Gaming Terms | 0.407 | 0.130 | 3.130 | 0.002 |
|  |  |  |  |  |
|  |  |  |  |  |
| N=191, 3820 total observations | | | |  |
